# Swallowing development in infants and toddlers with spinal muscular atrophy following therapy compared to healthy controls: the prospective controlled DySMA trial

**DOI:** 10.1186/s13023-026-04227-3

**Published:** 2026-02-16

**Authors:** Jana Zang, Charlotte Dumitrascu, Julia Glinzer, Deike Weiss, Jonas Denecke, Christina Pflug, Almut C. Niessen, Paula Steffens, Jessika Johannsen

**Affiliations:** 1 Department of Voice, Speech and Hearing Disorders, University Dysphagia Center, University Medical Center Hamburg-Eppendorf, Hamburg, Germany; 2 Institute of Health Sciences, University of Luebeck, Ratzeburger Allee 160, 23562 Luebeck, Germany; 3 Department of Pediatrics, University Medical Center Hamburg-Eppendorf, Hamburg, Germany

**Keywords:** Neuromuscular disease, Deglutition, Dysphagia, Feeding disorders

## Abstract

**Background:**

Swallowing development is a crucial outcome measure for evaluating the effectiveness of disease-modifying therapies (DMT) in children with spinal muscular atrophy (SMA). However, data on this aspect remain limited due to a lack of assessment tools. This study aimed to evaluate swallowing development in infants and toddlers with SMA, compare it with healthy controls (HC), and investigate the influence of initial symptom status at start of DMT and *SMN2* copy number.

**Methods:**

An observational study was conducted with infants and toddlers diagnosed with SMA at a single neuropediatric center and a HC group. Swallowing development was primarily assessed using the DySMA scale. Group differences and the impact of initial symptom status and *SMN2* copy number on swallowing were analyzed using linear mixed-effects models.

**Results:**

The study included 127 infants and toddlers, 35 with SMA (0–36 months, 19 girls), who started DMT either presymptomatically (*n* = 18) or symptomatically (*n* = 17), with two (*n* = 26) or three (*n* = 9) *SMN2* copies, predominantly receiving Onasemnogene abeparvovec alone (*n* = 26), and a healthy control group (*n* = 92, 0–23 months, 34 girls). Children with SMA displayed significantly different swallowing development trajectories (-0.06 DySMA points by months of life, *p*=.06; 95% CI -0.13 to 0.01) compared to HC (+ 0.45 DySMA points by months of life, *p*<.01, 95% CI 0.25 to 0.65). Notably, those treated presymptomatically demonstrated swallowing progress comparable to HC up to 15–17 months but experienced a decline thereafter (18–24 months; β = 8.22, *p*<.001; 95% CI 4.20 to 12.26). The group with three *SMN2* copies exhibited a developmental pattern similar to HC up to the age of 24 months.

**Conclusion:**

Higher *SMN2* copy number and presymptomatic DMT initiation were associated with similar early swallowing development compared to HC. Long-term studies stratifying swallowing development by *SMN2* copy number are warranted.

**Supplementary Information:**

The online version contains supplementary material available at 10.1186/s13023-026-04227-3.

## Introduction

Spinal muscular atrophy (SMA) is a rare inherited neurodegenerative disease caused by biallelic deletions or mutations in the survival motor neuron 1 (*SMN1*, MIM 600354). This leads to reduced production of the *SMN* protein and degeneration of lower motor neurons [1, 2].

Historically, SMA was classified into clinical types (0 to 4) based on symptom onset and motor function, with a lower number indicating greater disease severity. Currently the number of copies of the *SMN*2 gene is considered the best-known disease modifier, with the earliest symptom onset and highest disease severity occurring with one to three *SMN*2 copies [3, 4]. The *SMN*2 gene produces low amounts of *SMN* protein [5, 6]. When untreated, the most severe form of SMA results in significant muscle wasting, respiratory failure, and ultimately death within the first two years of life [7–9]. The introduction of newborn screening for SMA combined with the availability of three disease-modifying therapies (DMT), which positively impact survival and motor development, have sparked discussions regarding new SMA phenotypes and the necessity for novel monitoring tools to assess disease progression in the context of early treatment [10].

Previous case studies of the rare SMA type 0 (most commonly 1 *SMN2* copy) describe severe dysphagia from birth and early mortality [11]. In type 1 (most commonly two *SMN2* copies), severe dysphagia affects swallowing safety [12, 13], while type 2 (most commonly 3 *SMN2* copies) is linked to milder symptoms like impaired chewing [14, 15]. It has been shown that initiating DMT after symptom onset leaves a high risk for swallowing disorders [12, 16, 17].

Systematic reviews of swallowing function in SMA [8, 18] provide an overview of the various diagnostic methods used. Most studies cite the presence of dysphagia or use of a feeding tube as an indicator of feeding and swallowing problems, without specifying clinical diagnoses, or employed oral intake scales to evaluate how a child was fed.

In the NUTURE study follow-up [19], children receiving presymptomatic Nusineren (2 *SMN2* copies, *n* = 15; 3 *SMN2* copies, *n* = 10), were assessed via repeated parent questionnaires for dysphagia at the ages of 1.9–4.8 years (2 copies) and 1.9–3.8 years (3 copies). Children with two *SMN2* copies showed higher mean swallowing-related scores, indicating greater impairment. While all children with three *SMN2* copies maintained full oral nutrition, five of 15 children with two copies required tube feeding. A retrospective analysis of the SPR1NT trial [20] confirmed age-appropriate swallowing development for all children following presymptomatic treatment with Onasemnogene abeparvovec (2 *SMN2* copies *n* = 14, evaluated up to 18 months of age; 3 *SMN2* copies *n* = 15, evaluated up to 24 months of age). However, no healthy control group was included. In a multicenter observational study [21] of 343 individuals with SMA, early initiation of Onasemnogene abeparvovec and higher *SMN*2 copy number resulted in better swallowing (measured by presence or absence of a feeding tube).

To detect slight deviations from normal development specific instruments are necessary. Initial approaches include the international consensus on bulbar assessment in SMA [22], and the development of the DySMA for infants and toddlers aged zero to 24 months [23]. The clinical tool, developed by the authors of this study, provides a score analogous to motor function assessments reflecting physiological development and pathological progression.

The primary objective of this study was to compare swallowing development in infants and toddlers with SMA to HC. Two main questions were investigated: (1) How does swallowing development differ between infants and toddlers with SMA (aged 0–24 months) receiving DMT and HC? (2) What influence do ‘initial symptom status at start of DMT’ and ‘*SMN2* copy number’ have on swallowing development in infants with SMA?

Based on the aforementioned studies [19–21] and a pilot study [23], we hypothesized that: (1) Infants and toddlers with SMA exhibit a different swallowing development trajectory compared to HC. (2) Presymptomatic initiation of DMT would predict better swallowing development than symptomatic start of therapy. (3) Infants and toddlers with two *SMN2* copies would differ from those with three copies in swallowing development.

## Methods

### Study design and participants

The prospective controlled observational DySMA study (including the DySMAnorm study) is registered with the German Clinical Trials Register (DRKS00029541; date of registration 04.07.2022). The local ethics committee approved the study (2022-100827_2-BO-ff). All parents gave written informed consent. The study was planned and reported in accordance with the STROBE checklist for observational studies [24].

HC were recruited for the DySMAnorm study [25] between April 2023 and March 2024 through maternity clinics, playgroups, and daycare centers. Inclusion criteria involved birth after the 37th gestational week and no developmental abnormalities, verified by routine pediatric visit documentation.

All children with genetically classified 5q-associated SMA with two or three *SMN2* copies were prospectively recruited from a single neuropediatric center. No additional inclusion or exclusion criteria were specified. Infants were classified as presymptomatic if they showed no clinical symptoms of SMA, such as muscle hypotonia and/or weakness, reduced or absent tendon reflexes, tongue fasciculations, or bulbar symptoms. Electrophysiological data were not included in the definition. Data collection commenced in September 2020 with symptomatic infants, with a broader range of items collected for this group. After completion of the DySMA in 2022, these data were retrospectively re-evaluated for this group. Presymptomatic infants were first included from October 2021 with the start of newborn screening Data collection was completed by September 2024. Data on swallowing from nine children have already been published [17, 23].

### Procedure

The protocol included an initial data collection one day prior to DMT initiation, followed by assessments at intervals of two, four, and six months until age 24 months, and a follow-up in the third year of life. Assessments aligned with regular pediatric neurology check-ups according to SMARTCare registry guidelines [2]. A missed measurement point was not an exclusion criterion.

Clinical swallowing assessment appointments were conducted by speech-language pathologists (SLP) experienced in pediatric feeding and swallowing disorders. The primary outcome ‘swallowing development’ was measured using the DySMA, which comprises two parts: a patient history section reported by parents and a clinician examination. The DySMA is available online at 10.3233/JND-230177 (see Supplementary Material sj-pdf-3 at the very bottom of the page). The total score ranges from zero to 35 points. Higher scores are better than lower scores, and highest scores are normally observed around 24 months in typically developing children without SMA. Confirmation of content validity has been published [23]. The convergent construct validity was confirmed in comparison to flexible-endoscopic evaluation of swallowing (FEES) in symptomatic children with SMA (ρ= − 0.859, 95%CI − 0.961 to − 0.550, *p*<.001) see Supplementary Table S1 and Fig. S1.

In addition, maximum mouth opening (MMO in mm), the number of cases with pneumonia, and the current feeding route (oral, tube feeding, NPO), were documented. Nutritional status was assessed using WHO 2006 weight-for-length (WFL) Z-scores, calculated based on sex-specific WHO Child Growth Standards. Oral intake was categorized using the CEDAS (Children’s Eating and Drinking Activity Scale) [26]; score range 1–6, highest score indicating *total oral age-appropriate intake*). Motor function was assessed during routine check-ups using the CHOP-INTEND (Children’s Hospital of Philadelphia Infant Test of Neuromuscular Disorders [27]; score range 0–64, higher scores indicating function) by physiotherapists.

### Outcomes

The primary outcome swallowing development (measured by DySMA) was defined as infants’ basic ability to drink milk at birth and their progression to more advanced skills. Pathological development was defined as unexpected bulbar dysfunction associated with swallowing or feeding disorders (e.g., tongue fasciculation, impaired secretion management, very limited food intake).

The timing of first DMT initiation (clinically presymptomatic vs. symptomatic) and *SMN2* copy number (2 vs. 3 copies) were identified as potential predictors for the DySMA outcomes.

The secondary outcome included tongue fasciculations, high-arched palate, feeding route, MMO, pneumonia, CEDAS, WFL Z-scores, and CHOP-INTEND.

### Statistical analysis

Data were tabulated in Excel (2016) and transferred to R statistical software version 4.4.2 (2024-10-31) [28] for analysis with the ‘lme4’ package [29]. Descriptive statistics included median and interquartile range (IQR) for ordinal variables or mean and standard deviation (SD) for metric variables. For model statistics, the data were pooled into seven age groups defined in the DySMAnorm study by Dumitrascu and colleagues [25]. For children with SMA, the follow-up age group of 25–36 months was included in the descriptive data analysis. The reporting of the models was structured following the LEVEL recommendations for reporting multilevel data and analyses [30]. The significance level was set at α = 0.05.

Three linear mixed-effects models were calculated with a random intercept for each individual case to account for repeated measurements. The models were estimated using the restricted maximum likelihood method.

To answer the first research question (SMA vs. HC), model 1 (M1) was fitted. The dependent variable was the DySMA score, the fixed effects included the `Group` (SMA or HC) and the interaction between age in months and the group.

Based on previous studies [19, 23] two separate linear mixed-effects models were fitted to investigate the influence of ‘initial symptom status’ and ‘number of *SMN2* copies’ on swallowing development. Model 2 (M2) examined the interaction between age groups and initial symptom status (presymptomatic or symptomatic) on DySMA scores. Model 3 (M3) assessed the interaction between age groups and the number of *SMN2* copies (2 or 3) on DySMA.

For model fit, the models were compared with the likelihood-ratio-test (LRT). Akaike information criterion (AIC) was used as goodness of fit statistic. Model assumptions, including the distribution of the dependent variable and random effects, normality of residuals, influence of outliers, and collinearity between fixed effects were considered. An initial linear mixed-effects null model (M0: lmer(DySMA ~ 1 + (1|Case)), which included only a random intercept for each case, was compared with all full models using the `lmer` function. M1: DySMA ~ Group + Age_months: Group + (1 | Case)). M2: DySMA ~ Age_group: Initial_symptom_status + (1 | Case). M3: DySMA ~ Age_group: SMN2_coNr + (1 | Case).

### Role of the funding source

This study was not funded.

## Results

### Cohort

**Fig. 1:**
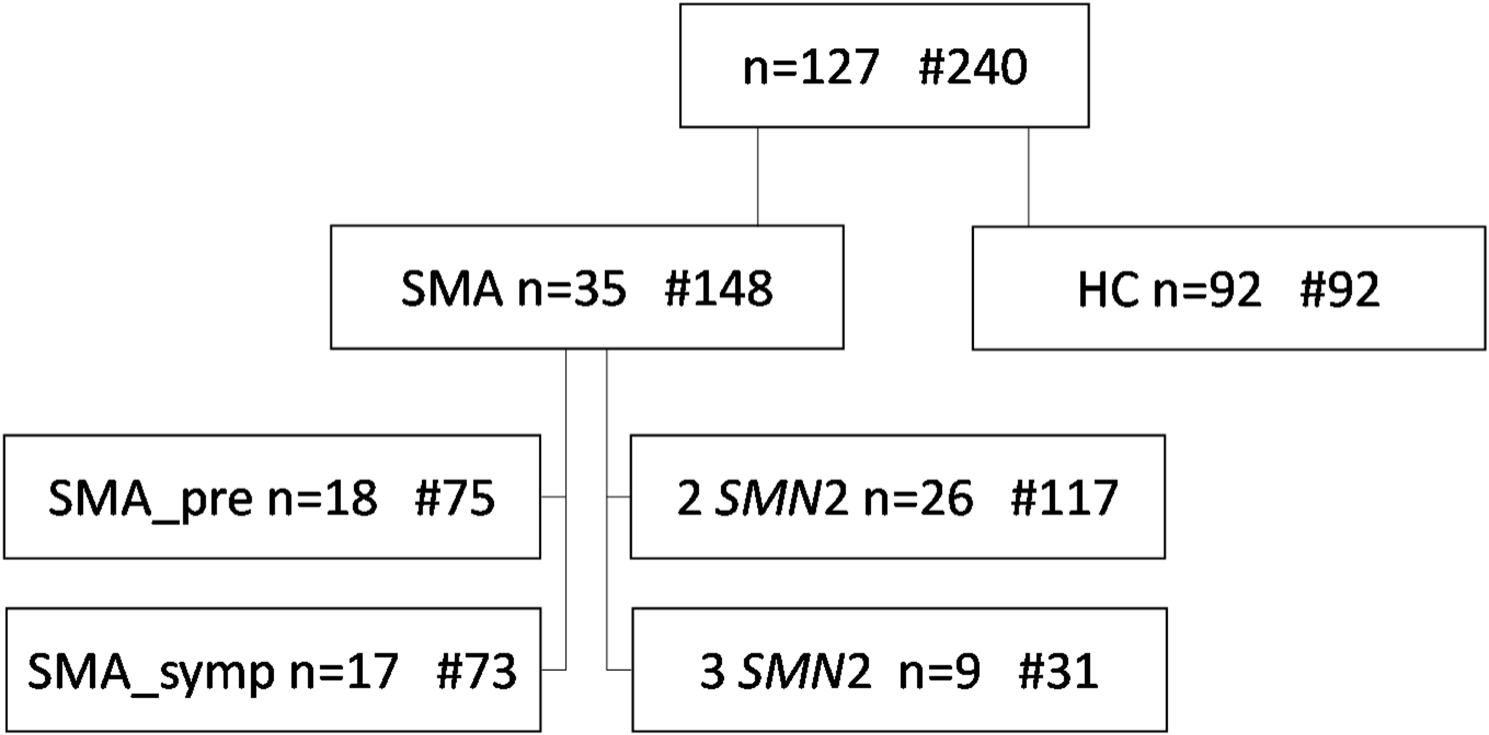
Overview of included cases and group classification. The number of examinations per group is indicated by a #. HC = healthy controls, pre = presymptomatic start of DMT, symp = symptomatic start of DMT, 2 *SMN*2 = 2 *SMN2* copies, 3 *SMN2* = *3 SMN2* copies. Please note that groups can be classified either by the initiation of DMT and by the number of *SMN2* copies

The DySMA study cohort comprised 127 infants and toddlers. A total of 36 infants were recruited for the SMA cohort; one family declined participation, resulting in 35 cases with SMA. Over the study period, the children were aged between zero and 35 months. For further analysis, the SMA cohort was divided into presymptomatically treated children (SMA_pre *n* = 18) and symptomatically treated children (SMA_symp *n* = 17), or by *SMN2* copy number (Fig. 1). Patient characteristics of the SMA sample are shown in Table 1. Included was one presymptomatic twin pair (2 *SMN2* copies). Since there were very few cases for Nusinersen and Risdiplam, it was not possible to divide the children into groups per drug.

A total of 148 assessments were conducted in the SMA cohort (M = 4.2 ± 1.79; range 1–8) see Fig. 1. Due to missing assessments resulting from children’s illnesses or scheduling difficulties, the number of measurements varied among the participants. The pooled measurement points into age groups, and the corresponding number of examinations per subgroup and age group are presented in Supplementary Table S2.

The HC group (*n* = 92), which included 34 girls (37%) and 58 boys aged between zero and 23 months, is described in detail in the DySMAnorm study [25].

**Table 1:** Clinical characteristics and disease modifying treatments of patients with SMA

	SMA (total) *n* = 35	SMA_pre *n* = 18	SMA_symp *n* = 17	2 SMN2 *n* = 26	3 SMN2 *n* = 9
**Gender**					
Girls n (%)	19 (54.3)	10 (55.6)	9 (52.9)	15 (57.7)	4 (44.4)
Boys n (%)	16 (45.7)	8 (44.4)	8 (47.1)	11 (42.3)	5 (55.6)
***SMN2*** **copy number**					
3 n (%)	9 (25.7)	7 (38.9)	2 (11.8)	0	9 (100)
2 n (%)	26 (74.3)	11 (61.1)	15 (88.2)	26 (100)	0
**Initial symptom status at start of first DMT**					
Presymptomatic	18 (51.4)	18 (100)	0	11 (42.3)	7 (77.8)
Symptomatic	17 (48.6)	0	17 (100)	15 (57.7)	2 (22.2)
**Age at start of treatment in days**					
Mdn (IQR)	34.0 (80)	25.5 (15)	98.0 (207)	34.5 (89)	27 (216)
Range	13–542	13–40	17–542	13–267	18–542
M (± SD)	92.29 (± 124.43)	26.11 (± 8.05)	162.35 (± 150.64)	79.42 (± 83.16)	129.44 (± 205.19)
**Type of DMT**					
Onasemnogene alone n (%)	26 (74.3)	16 (88.9)	10 (58.8)	19 (73.1)	7 (77.8)
Nusinersen alone	2	1	1	1	1
Risdiplam alone	1	1	0	1	0
Nusinersen followed by Onasemnogene	3	0	3	3	0
Risdiplam followed by Onasemnogene	3	0	3	2	1

pre = presymptomatic start of DMT, symp = symptomatic start of DMT, 2 *SMN2* = 2 *SMN*2 copies, 3 *SMN2* = 3 *SMN2* copies; Mdn=Median, IQR=interquartile range, M=Mean, SD=standard deviation

### Swallowing-related outcome

The mean values across all measurements for the primary outcome DySMA and the secondary outcomes are presented per group in Table 2. In the HC group the DySMA ranged from a minimum of 21 to the maximum of 35, with the lowest scores occurring in the youngest age groups (0–2 and 3–5 months) and the highest score from 15 to 17 months onward. For the total group of children with SMA, DySMA scores ranged from zero to 34, with no child reaching the maximum score of 35. The median DySMA scores of children with SMA (Mdn = 25; IQR 12) are lower than those of the HC group (Mdn = 31; IQR 6). The lowest values were seen in the symptomatic group (Mdn = 17; IQR 23) and in infants and toddlers with two *SMN*2 copies (Mdn = 23; IQR 14), Table 2 and Supplementary Fig. S2.

Median CEDAS scores were not sufficient to demonstrate differences between the groups in this study, though there were descriptive differences in the min-max ranges. WFL Z-scores and median MMO differed significantly between groups. (Table 2). All children with three *SMN2* copies maintained full oral nutrition. In the presymptomatic group, 17 of 18 children received full oral nutrition, with one receiving intermittent nasogastric nutrition during pneumonia. CHOP-INTEND values were only available for children with SMA. The median values for the presymptomatic group and for the group with three *SMN2* copies were higher than those in other SMA groups (Supplementary Fig. S3). The relationship between motor function and swallowing development is presented in Supplementary Table S3 and Fig. S4.

Tongue fasciculations occurred only in children with two *SMN*2 copies, even after presymptomatic start of DMT, with none observed in those with three copies up to 35 months. A high-arched palate was present in all groups, most frequently in children with two SMN2 copies (Table 2).

**Table 2:** Primary and secondary (swallowing related) outcomes across multiple assessment time points stratified by HC, children with SMA and subgroups

	HC *n* = 92	SMA (total) *n* = 35	SMA_pre *n* = 18	SMA_symp *n* = 17	2 *SMN2* *n* = 26	3 *SMN2* *n* = 9
**DySMA [0–35]**						
Mdn (IQR)	31.0 (6)	25.0 (12)	27.0 (3)	17.0 (23)	23.0 (14)	28.0 (7)
Range	21–35	0–34	14–34	0–34	0–33	21–34
M (± SD)	29.1 (± 3.55)	21.28 (± 9.8)	26.43 (± 3.90)	16.0 (± 11.15)	19.26 (± 9.91)	28.9 (± 3.82)
**CEDAS** [1–6]						
Mdn (IQR)	6 (0)	6 (1)	6 (0)	5 (4)	5 (4.0)	6.0 (0)
Range	6–6	1–6	3–6	1–6	1–6	5–6
**WFL Z-score**						
Mdn (IQR)	0.96 (2.1)	− 0.14 (2.3)	0.14 (2.5)	− 0.43 (2.4)	− 0.59 (2.5)	0.33 (1.7)
Range	-2.9- 3.6	-3.8- 3.7	-3.6- 3.7	-3.8- 3.7	-3.8- 3.7	-1.6- 3.3
M (± SD)	1.0 (± 1.55)	− 0.13 (± 1.71)	0.12 (± 1.78)	− 0.37 (± 1.62)	− 0.32 (± 1.76)	0.57 (± 1.26)
**MMO**						
Mdn (IQR)	32.0 (7)	25.0 (6)	26.0 (6)	24.5 (8)	24.0 (6)	28.0 (8)
Range	15–40	17–38	17–36	18–38	17–35	20–38
M (± SD)	30.9 (± 4.61)	25.25 (± 4.70)	25.37 (± 4.31)	25.03 (± 5.4)	24.0 (± 4.12)	27.89 (± 4.83)
**CHOP-INTEND** [0–64]						
Mdn (IQR)	.	40.5 (25)	47.0 (28)	37.0 (22)	38.0 (25)	54.0 (28)
Range	.	1–64	12–64	1–62	1–64	29–64
M (± SD)	.	39.81 (± 15.64)	44.3 (± 15.15)	35.4 (± 14.97)	37.76 (± 15.30)	50.26 (± 13.32)
**Feeding Route**						
Oral n (%)	92 (100)	27 (77.1)	17 (94.4)	9 (52.9)	18 (69.2)	9 (100)
Tube (NPO)	0	8 (7)	1*(0)	8 (7)	8 (7)	0
**Tongue fasciculation**						
n (%)	0	22 (62.9)	8 (44.4)	14 (82.2)	22 (84.6)	0
**High-arched palate**						
n (%)	2 (2.2)	19 (54.3)	6 (33.3)	12 (70.6)	18 (69.2)	1 (11.1)
**Pneumonia**						
n	0	10	2	10	10	0

SMA_pre = presymptomatic start of DMT. SMA_symp = symptomatic start of DMT. 2 *SMN*2 = 2 S*MN*2 copies. 3 *SMN*2 = 3 *SMN*2 copies; Mdn=Median. IQR=interquartile range. M=Mean. SD=standard deviation. HC=healthy controls. WFL=weight-for-length. MMO=Maximal mouth opening. NPO = Nil per os

#### Comparison with healthy controls

**Fig. 2:**
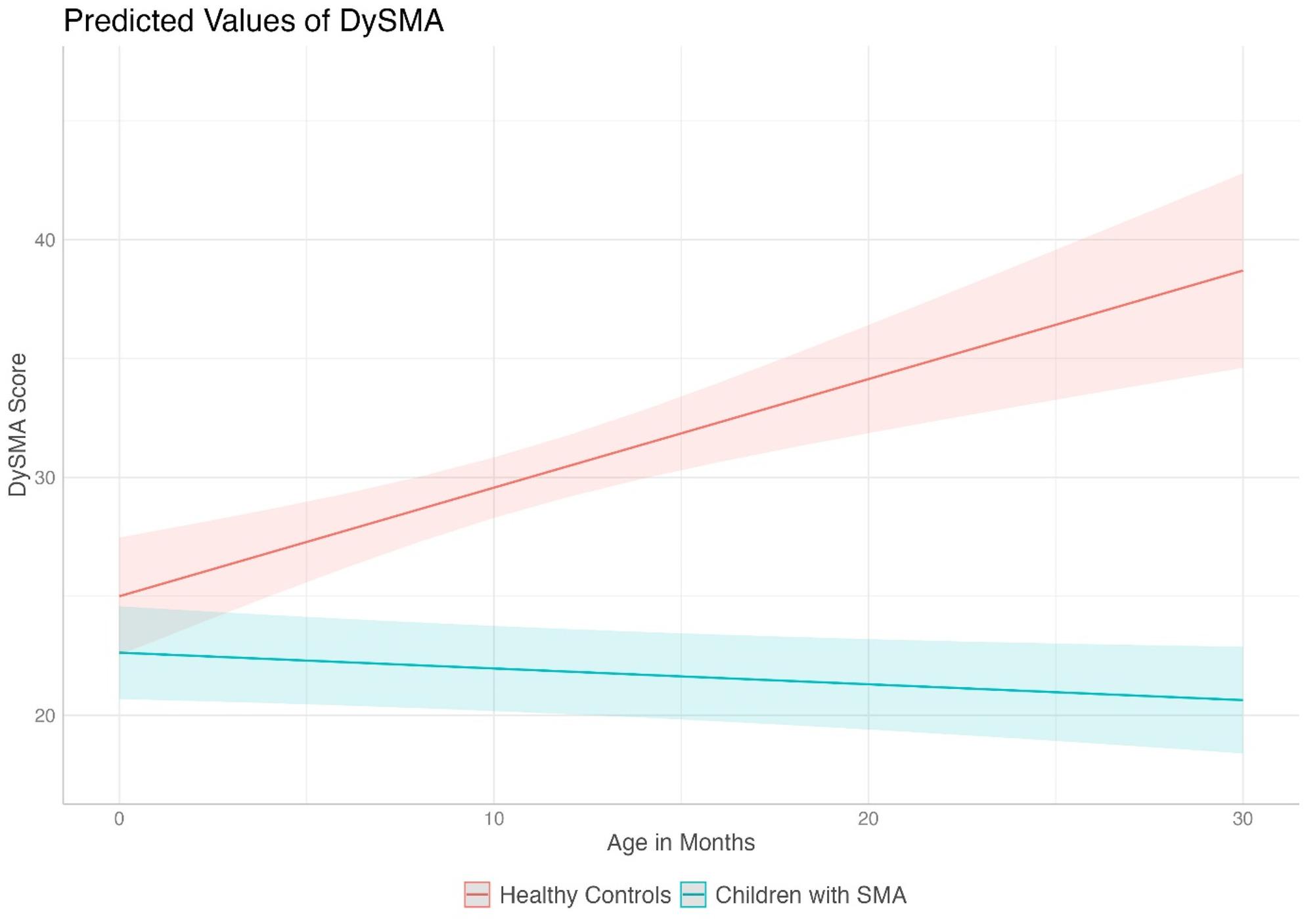
Comparison of swallowing development in HC and children with SMA - Predicted values of DySMA; random-intercept linear mixed-effects model for comparison of swallowing development in healthy controls (HC, *n* = 92) and SMA (*n* = 35). Note: These plotted values are model predictions, not the actual observed values, these are provided in Table 2 and in Supplementary Fig. S2

M1 indicated a significant increase of 0.45 DySMA points with each month of life (*p*<.001) in HC. In the SMA cohort, the DySMA decreased on average by 0.06 points per month (*p*=.067) and tended to stagnate (Table 3). Figure 2 visualizes the predicted values of the model.

**Table 3:** Results of the random-intercept linear mixed-effects model (M1) for comparison of swallowing development in HC and children with SMA

Variables	Coefficient (SE)	*p*-value	95% CI
*Fixed Effects*			
Intercept	25.00 (1.25)	< 0.01*	22.55–27.44
Group_SMA	-2.36 (1.59)	0.139	-5.48–0.74
Group_HC: Age_months	0.45 (0.10)	< 0.01*	0.25–0.65
Group_SMA: Age_months	-0.06 (0.03)	0.06	-0.13–0.01
*Model Information*			
AIC	1475.23	.	.
BIC	1496.11	.	.
Number of observations	240	.	.
Number of Groups	127	.	.

SE= Standard Error (SE), AIC= Akaike Information Criterion, BIC= Bayesian Information Criterion 95%CI = 95% Confidence Interval. The likelihood ratio test comparing the null model (LogLik(M0)= -761.62) to the full model (LogLik(M1)= -734.11) indicated a significant improvement (χ²(3) = 55.02, *p* < .001)

#### Initial symptom status

M2 demonstrated that the SMA_pre group initially exhibited a swallowing development comparable to HC, with a consistent increase in DySMA scores with age. However, from 15 to 17 months, a slight decrease was observable, followed by a significant drop in the 18–24 months age group (β = 8.22; *p*<.001), diverging substantially from HC.

**Fig. 3:**
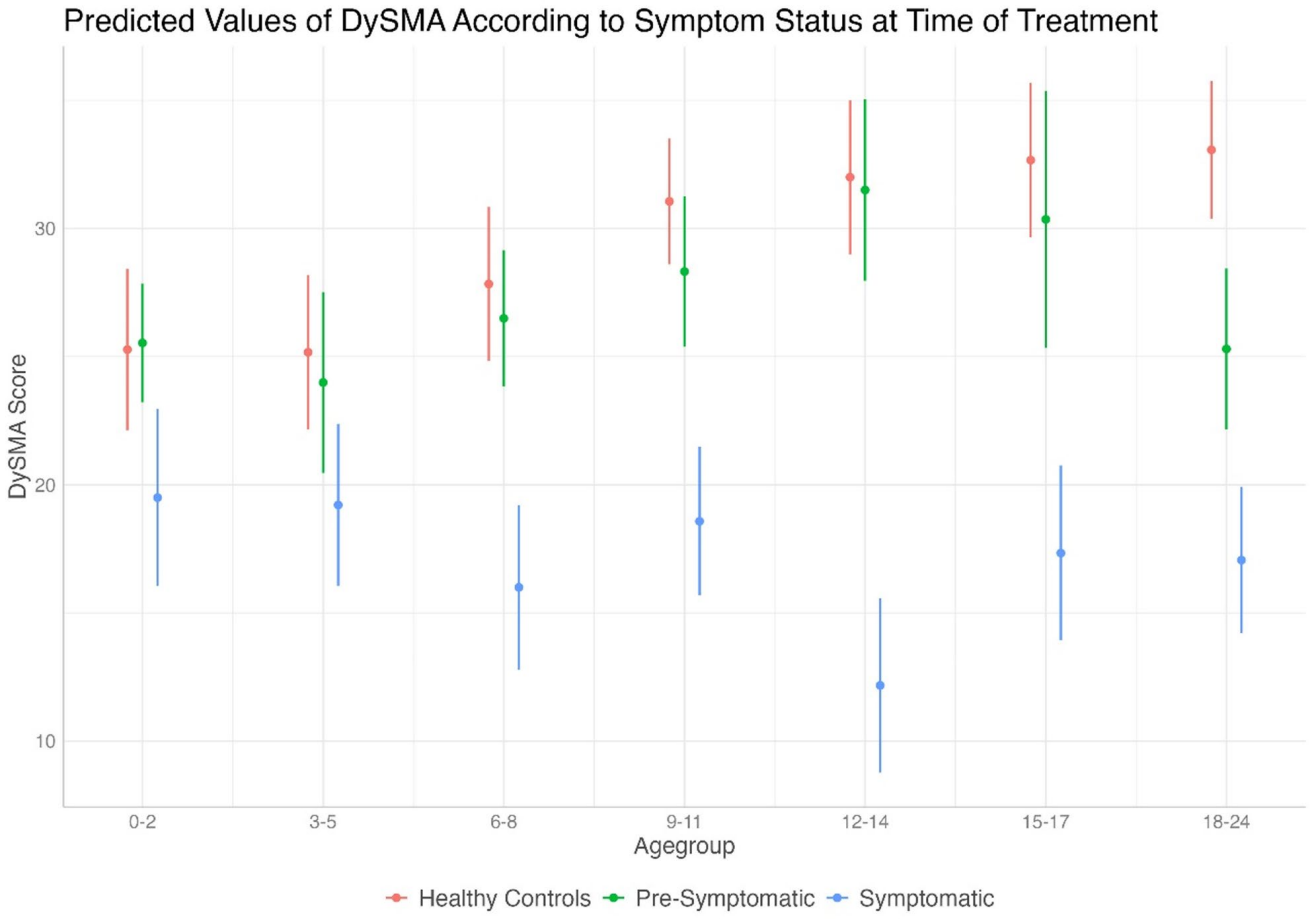
Influence of initial symptom status on swallowing development in children with SMA - Predicted values of DySMA in the random-intercept linear mixed-effects model for influence of initial symptom status (pre-symp, *n* = 18 or symp, *n* = 17*)* on swallowing development in children with SMA compared to healthy controls (HC, *n* = 92). Note: These plotted values are model predictions, not the actual observed values, these are provided in Table 2 and in Supplementary Fig. S2

The SMA_symp group showed little alteration in swallowing with increasing age, with DySMA scores significantly lower than those of HC. Furthermore, a significant decrease in scores was observed at 12–14 months (β = -4.89; *p* = .004) (Fig. 3 and Supplementary Table S4).

#### SMN2 copy number

**Fig. 4:**
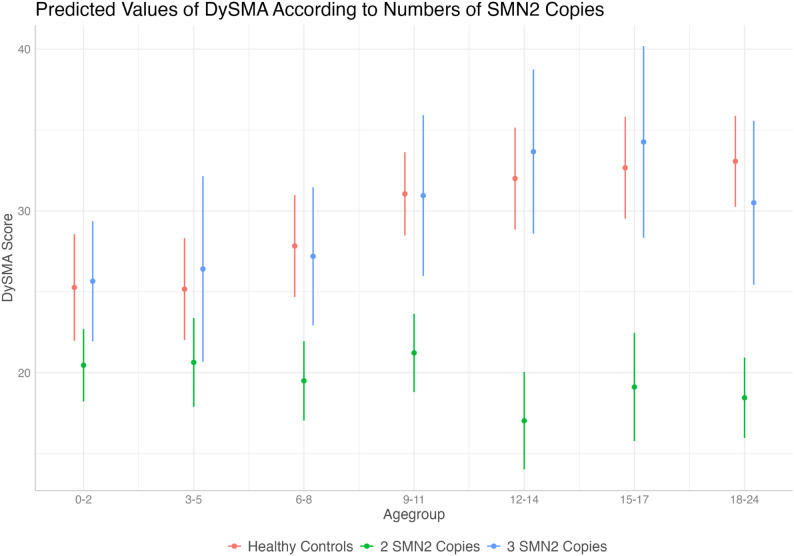
Influence of *SMN2* copy number on swallowing development in children with SMA -Predicted values of DySMA in the random-intercept linear mixed-effects model for influence of *SMN2* copy number (2, *n* = 26 or 3, *n* = 9) on swallowing development in children with SMA compared to healthy controls (HC, *n* = 92). Note: These plotted values are model predictions, not the actual observed values, these are provided in Table 2 and in Supplementary Fig. S2

M3 investigated the impact of *SMN2* copy number on swallowing development. The group with two *SMN2* copies showed significant negative effects across all age groups (*p* <.001), indicating significantly poorer swallowing development than the HC group, with the most pronounced effect observed in the 12–14 months age group (β = -13.46; *p*<.001) (Fig. 4 and Supplementary Table S5).

Conversely, no significant differences were found for infants with three *SMN2* copies, compared to HC in any age group, suggesting that while two SMN2 copies correlate with poorer swallowing development, three SMN2 copies appear positively associated, aligning with the development trajectory of HC over the first two years.

## Discussion

Our findings demonstrate that infants and toddlers with SMA do not develop swallowing skills comparable to their healthy peers, even when they receive early DMT. While presymptomatic initiation of DMT significantly predicts better swallowing development compared to starting DMT after symptom onset, it becomes evident that even these presymptomatically treated children do not reach the same levels of swallowing capability as HC by 15–17 months. Notably, infants and toddlers with three *SMN2* copies exhibit significantly better swallowing development compared to those with two copies, showing a trajectory that closely aligns with HC up to 24 months.

Regarding symptomatically treated children, our results align with previous studies indicating that they did not demonstrate significant improvement in swallowing outcomes [12, 17]. In terms of presymptomatic treatment, our results partially go in line with Shell et al. [20], who reported normal swallowing function for infants with presymptomatic start of treatment (Onasemnogene) (a) up to 18 months of age for two *SMN2* copies and (b) up to 24 months for children with three *SMN2* copies. However, the absence of a control group, the period of analysis (up to 18 or 24 months of age), and the definition of normal swallowing might be viewed critically. Future observations of presymptomatically treated children beyond the second year of life will be crucial in understanding the evolution of their swallowing development.

The influence of initial symptom status and *SMN2* copy number aligns with findings from past studies [19, 21] that reported a positive effect of early treatment and higher *SMN2* copy number on both swallowing and motor function. Weiss and colleagues [21] propose that individual production of functional *SMN* protein, determined by *SMN2* copy number, could enhance the efficacy of drug therapy.

In our cohort, differences between children with two versus three *SMN2* copies were evident in simple bulbar markers, such as MMO or tongue fasciculations. Notably, none of the children with three *SMN2* copies exhibited tongue fasciculations by 36 months and their MMO measurements were higher compared to those with two *SMN2* copies. Previous studies with untreated cohorts have indicated that children with SMA type 2 (most commonly 3 *SMN2* copies) typically experience swallowing difficulties that develop later or differently than those with type 1 (most commonly 2 *SMN2* copies).

### Limitations

The DySMA study carries limitations. Convergent construct validity is limited as FEES data were available for only a small group of symptomatic children. Instrumental swallowing diagnostics are considered the gold standard for assessing underlying swallowing physiology and pathophysiology but ethically, performing FEES or VFSS on HC or asymptomatic infants with SMA is not feasible. Additionally, while the goal of a clinical swallowing examination is to assess whether and to what extend a swallowing disorder is present, the DySMA establishes the trajectory of swallowing development and identifies deviations from expected pattens.

Another critical point is the observation period. HC data were only collected up to 24 months, while some children with SMA had follow-ups extending to 36 months. Further development and the persistence of group differences, deterioration, or improvement remain unclear. The small cohort size and single-center design warrant cautious interpretation, despite this study’s rare disease cohort being relatively large compared to previous studies focusing on primary swallowing outcomes.

It is important to note that the SMA participants in our study were classified as clinically presymptomatic, raising questions about whether they may have already exhibited electrophysiological abnormalities. Currently, there is no objective method to exclude bulbar involvement. Assessing swallowing development in presymptomatically treated cohorts requires careful evaluation of minor deviations from the normal development, as the mere ‘absence’ of dysphagia symptoms is insufficient for meaningful comparisons.

### Future directions

The developmental trajectory of swallowing skills in infants and toddlers makes it difficult to compare skills before and after treatment, as increasing age typically correlates with improved skills. Therefore, future studies in infancy must consider swallowing development as a process and in comparison to healthy controls. Beyond developmental effects, additional methodological challenges complicate the interpretation and comparison of swallowing outcomes across studies. For comparison with previous study results, a direct transfer of SMA types to *SMN2* copy number is problematic, as the type classification is based on motor milestones, and the *SMN2* copy number is not clear for each reported study cohort. Regarding the swallowing outcome, groups with a high proportion of three *SMN2* copies may achieve better results. In the future, it will be interesting to make group comparisons based on the *SMN*2 copy number.

A critical aspect to explore is whether progress in motor function corresponds to swallowing development. Previous research on symptomatic children reported improvements in motor function following treatment; however, these improvements did not consistently translate into enhanced bulbar function [12, 17]. To date, it is unclear how early brainstem motor neurons and cranial nerves are affected in infants with SMA, although tongue fasciculations have long been recognized as an initial symptom of early-onset SMA [7, 31, 32]. Our data showed a weak to moderate correlation between DySMA and CHOP-INTEND scores, depending upon classification of SMA groups. While this study did not aim to compare motor to bulbar function directly, evaluating this in future studies may provide valuable insights.

Two hypotheses arise regarding discrepancies between the development of motor and swallowing functions: (1) Bulbar impairment may manifest earlier than motor impairment, going undetected before the start of DMT. (2) Current therapies do reach the cranial nerve nuclei (bulbar) less effectively than the anterior horn cells of the spinal cord.

In summary, our findings underline the necessity of monitoring swallowing development alongside motor function in both clinical practice and research settings. We recommend using the DySMA as a systematic tool for data collection from birth to 36 months. Additionally, we advise evaluating bulbar outcomes based on *SMN2* copy number during the first years of life to enhance understanding and treatment strategies.

## Supplementary Information

Below is the link to the electronic supplementary material.

Supplementary Material 1

## Data Availability

Data are tabulated in the manuscript. Further data are available from the corresponding author upon reasonable request.

## Declarations

### Data sharing

The study data are reported in this manuscript and its supplement. Further data are available from the corresponding author on reasonable request from the moment of publication.
