# Supplementary material for: Swallowing development in infants and toddlers with spinal muscular atrophy following therapy compared to healthy controls: the prospective controlled DySMA trial
Source: Orphanet J Rare Dis. 2026 Feb 16;21:105. doi: 10.1186/s13023-026-04227-3 (PMC13015008; doi:10.1186/s13023-026-04227-3)
Supplement: Supplementary file 1 — Supplementary Material 1 [file 13023_2026_4227_MOESM1_ESM.docx]

Zang et al.

Supplementary Material:

Swallowing development in infants and toddlers with spinal muscular atrophy following therapy compared to healthy controls: the prospective controlled DySMA trial

*Convergent constrict validity of the DySMA*

In order to confirm the convergent construct validity of the DySMA, in a small group in whom a pharyngeal swallowing disorder was suspected or clinically present, the Penetration-Aspiration Scale (PAS, according to Rosenbek [1]) was assessed using FEES (see Zang et al. [2], for the full protocol). The PAS ranges from 1 (material does not enter the airway) to 8 (material enters the airway, passes below the vocal folds, and no effort is made to eject it), with higher scores indicating greater severity. The convergent construct validity was analyzed using nonparametric Spearman correlation between PAS scores and DySMA scores. Interpretation was based on the conventional approach of interpreting correlation coefficient in medical research: 0·4-0·69 (moderate correlation), 0·70-0·89 (strong correlation), and 0·90-1·99 (very strong correlation), and based on the 95%CI [3].

**Supplementary Table S1 Patient characteristics of children with SMA with FEES exams for comparison with DySMA**

| Case | #Exam | Age in months | Sex | DySMA [0-35] | PAS [1-8] |
| --- | --- | --- | --- | --- | --- |
| 1 | 1 | 2 | b | 15 | 3 |
| 2 | 1 | 6 | b | 1 | 8 |
|  | 2 | 15 |  | 1 | 8 |
| 3 | 1 | 9 | g | 2 | 5 |
|  | 2 | 15 |  | 1 | 5 |
| 4 | 1 | 9 | b | 0 | 6 |
|  | 2 | 14 |  | 1 | 3 |
| 5 | 1 | 7 | b | 13 | 3 |
|  | 2 | 14 |  | 12 | 4 |
| 6 | 1 | 7 | g | 22 | 1 |
|  | 2 | 13 |  | 19 | 1 |
| 7 | 1 | 9 | g | 19 | 2 |

All children in this group received symptomatic DMT and had two *SMN*2 copies and clinical signs of dysphagia. Five children had two FEES exams, #exam indicates whether it was the first or the second examination; PAS=Penetration-aspiration Scale.

There was a strong negative correlation (ρ= -·859) between PAS (with higher values indicating greater severity (Table S1 and Fig. S1) DySMA scores with a 95% confidence interval ranging from - ·961 to - ·550 (p<·001). Note that there is no other instrument available measuring the construct of “swallowing development”. Strictly speaking, by PAS (or FEES), the construct of dysphagia or laryngeal penetration and aspiration is measured. As can be seen in the low DySMA scores (Table S1), all children had DySMA scores of less than or equal to 22, thus indicating pathological development.


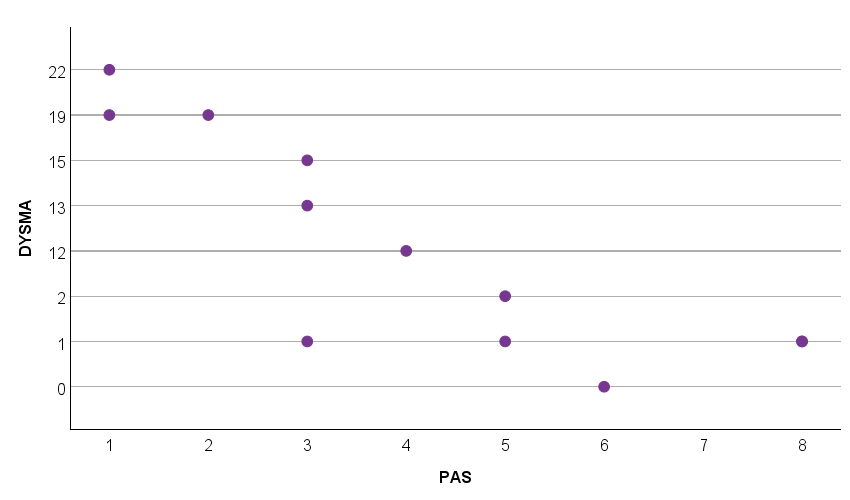


**Supplementary Fig. S1** Correlation of DySMA with FEES-determined PAS scores of 17 examinations in ten children with SMA. Higher DySMA values (0-35) indicate better swallowing development. Since this group only reaches a maximum value of 22, the y-axis only includes the values 0-22. PAS (1-8): higher scores expressing a higher degree of severity.

***Number of assessments***

**Supplementary Table S2: Number of pooled measurement points by age group stratified by subgroup**

| Age group | 0-2 | 3-5 | 6-8 | 9-11 | 12-14 | 15-17 | 18-24 | 25-36 | Total |
| --- | --- | --- | --- | --- | --- | --- | --- | --- | --- |
| SMA (total) | 35 | 13 | 20 | 19 | 11 | 8 | 21 | 21 | 148 |
| SMA_pre | 29 | 5 | 13 | 9 | 5 | 2 | 8 | 4 | 75 |
| SMA_symp | 6 | 8 | 7 | 10 | 6 | 6 | 13 | 17 | 73 |
| 2 *SMN*2 | 25 | 11 | 15 | 16 | 8 | 6 | 17 | 19 | 117 |
| 3 *SMN*2 | 10 | 2 | 5 | 3 | 3 | 2 | 4 | 2 | 31 |
| HC | 11 | 12 | 12 | 18 | 12 | 12 | 15 | 0 | 92 |
| Total | 46 | 25 | 32 | 37 | 23 | 20 | 36 | 21 | 240 |

The SMA group is divided into presymptomatic (SMA_pre) and symptomatic (SMA_symp), as well as by the number of SMN2 copies (2 *SMN*2, 3 S*MN*2). HC = healthy controls.

***Swallowing Development***


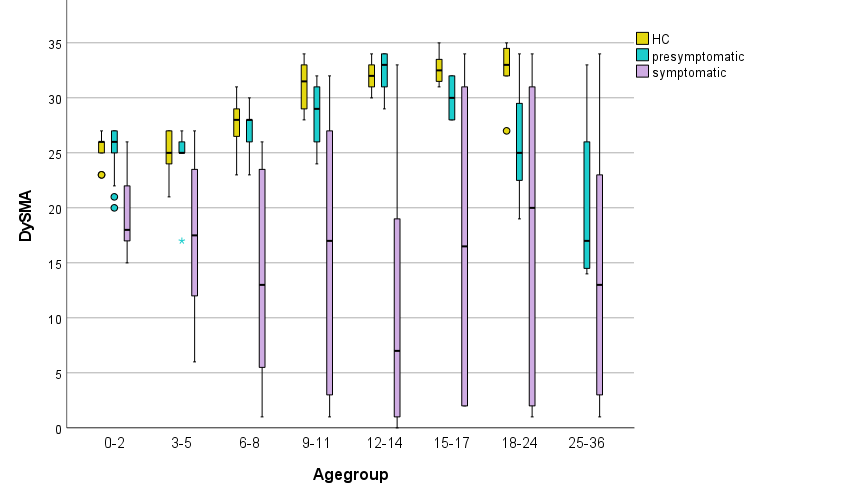


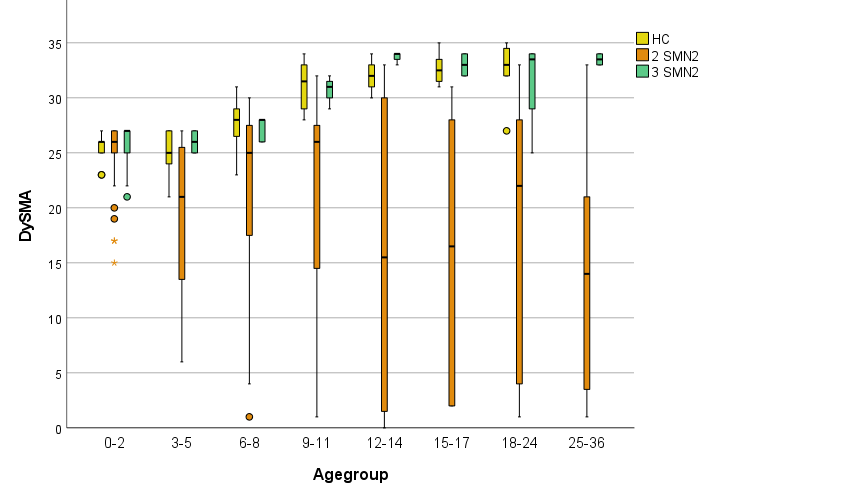


**Supplementary Fig. S2** Boxplots per age group for the observed DySMA values (swallowing development) for children with SMA and healthy controls. **a)** Grouped by initial symptom status (presymptomatic, n=18 or symptomatic, n=17*)* and **b)** grouped by SMN2 copy number (2 S*MN*2, n=26 or 3 *SMN2*, n=9). HC=Healthy controls (n=92)), DySMA ranges from 0 to 35, with a higher score indicating a better outcome.

***Motor Function***

**Supplementary Fig. S3** Boxplots per age group for the observed CHOP-INTEND values (motor function) in children with SMA**.** a) Grouped by initial symptom status (presymptomatic, n=18 or symptomatic, n=17*)* and b) grouped by SMN2 copy number (2 *SMN*2, n=26 or 3 *SMN*2, n=9). CHOP-INTEND ranges from 0 to 64, with a higher score indicating a better outcome.

***Motor function vs. swallowing development***

**Supplementary Table S3 Correlation (Spearman's Rho) between CHOP-INTEND and DySMA in children with SMA**

|  | Spearman-Rho | p-Value | Strength of correlation |
| --- | --- | --- | --- |
| SMA (total) | ·496 | <·001* | moderate |
| SMA_pre | ·350 | ·008* | weak |
| SMA_symp | ·464 | <·001* | moderate |
| 2 *SMN*2 | ·427 | <·001* | moderate |
| 3 *SMN*2 | ·409 | ·082 | moderate |

pre = presymptomatic start of DMT, symp = symptomatic start of DMT, 2 *SMN*2 = 2 *SMN2* copies, 3 *SMN2* = *3 SMN2* copies. Significance was tested two-tailed.

**Supplementary Fig. S4** Scatter plot to visualize correlation of CHOP-INTEND and DYSMA in children with SMA**.** a) Grouped by initial symptom status (presymptomatic, n=18 or symptomatic, n=17) and b) grouped by SMN2 copy number (2 SMN2, n=26 or 3 SMN2, n=9). CHOP-INTEND ranges from 0 to 64, with a higher score indicating a better outcome. DySMA ranges from 0 to 35 with a higher score indication a better development.

***Mixed-Effects Models***

**Supplementary Table S4 Results of the final random-intercept linear mixed-effects model (M2) for the influence of “initial symptom status” on swallowing development in children with SMA.**

| **Variables** | **Coefficient (SE)** | **p-value** |  | **95% CI** |
| --- | --- | --- | --- | --- |
| *Fixed Effects* | | | | |
| Intercept | 17·06 (1·44) | < ·001** |  | 14·33 – 19·76 |
| 0-2:HC | 8·20 (2·14) | < ·001** |  | 4·17 – 12·27 |
| 3-5:HC | 8·10 (2·09) | < ·001** |  | 4·16 – 12·07 |
| 6-8:HC | 10·78 (2·09) | < ·001** |  | 6·83 – 14·73 |
| 9-11:HC | 13·99 (1·90) | < ·001** |  | 10·41 – 17·59 |
| 12-14:HC | 14·93 (2·09) | < ·001** |  | 10·99 – 18·90 |
| 15-17:HC | 15·60 (2·09) | < ·001** |  | 11·66 – 19·57 |
| 18-24:HC | 16·00 (1·98) | < ·001** |  | 12·27 – 19·75 |
|  |  |  |  |  |
| 0-2:pre_symp | 8·46 (1·86) | < ·001** |  | 4·97 – 11·98 |
| 3-5:pre_symp | 6·92 (2·29) | ·002* |  | 2·62 – 11·23 |
| 6-8:pre_symp | 9·42 (1·97) | < ·001** |  | 5·71 – 13·14 |
| 9-11:pre_symp | 11·25 (2·07) | < ·001** |  | 7·37 – 15·15 |
| 12-14:pre_symp | 14·43 (2·30) | < ·001** |  | 10·12 – 18·75 |
| 15-17: pre_symp | 13·28 (2·92) | < ·001** |  | 7·83 – 18·75 |
| 18-24: pre_symp | 8·22 (2·15) | < ·001** |  | 4·20 – 12·26 |
|  |  |  |  |  |
| 0-2:symp | 2·43 (1·73) | ·162 |  | -0·79 – 5·68 |
| 3-5:symp | 2·15 (1·56) | ·171 |  | -0·76 – 5·08 |
| 6-8:symp | -1·06 (1·62) | 5·13 |  | -4·08 – 1·97 |
| 9-11:symp | 1·51 (1·44) | ·293 |  | -1·16 – 4·22 |
| 12-14:symp | -4·89 (1·68) | ·004* |  | -8·02 - -1·74 |
| 15-17:symp | 0·27 (1·77) | ·876 |  | -3·02 – 3·59 |
| *Model Information* |  |  |  |  |
| AIC | 1250·37 | .. | .. | .. |
| BIC | 1328·32 | .. | .. | .. |
| Num. obs. | 219 | .. | .. | .. |
| Number of Groups | 127 | .. | .. | .. |

SE= Standard Error, AIC= Akaike Information Criterion, BIC= Bayesian Information Criterion 95%CI= 95% Confidence Intervall. Note: the fixed-effect model matrix was rank deficient, leading to the removal of the last colum ‘18-24:symp’ because it did not provide any additional information. The likelihood ratio test indicated a highly significant improvement in fit with inclusion of fixed-effects (χ²(20) = 186·74, p < ·001; LogLik(M0)= -695·56; LogLik(M2)=, -602·19).

**Supplementary Table S5** **Results of the final random-intercept linear mixed-effects model (M3) for the influence of “SMN2 copy number” on swallowing development in children with SMA**

| **Variables** | **Coefficient (SE)** | **p-value** |  | **95% CI** |
| --- | --- | --- | --- | --- |
| *Fixed Effects* | | | | |
| Intercept | 30·50 (2·58) | < ·001* |  | 25·67 – 35·29 |
| 0-2:HC | -5·23 (3·06) | ·089 |  | -10·95 – 0·53 |
| 3-5:HC | -5·33 (3·02) | ·079 |  | -10·98 – 0·35 |
| 6-8:HC | -2·66 (3·02) | ·378 |  | -8·32 – 3·02 |
| 9-11:HC | 0·55 (2·88) | ·848 |  | -4·82 – 5·97 |
| 12-14:HC | 1·49 (3·02) | ·621 |  | -4·15 – 7·19 |
| 15-17:HC | 2·16 (3·02) | ·475 |  | -3·48 – 7·85 |
| 18-24:HC | 2·56 (2·93) | ·384 |  | -2·92 – 8·09 |
|  |  |  |  |  |
| 0-2:coNr 2 | -10·03 (2·80) | <·001** |  | -15·30 - -4·78 |
| 3-5:coNr 2 | -9·86 (2·91) | <·001** |  | -15·30 - -4·36 |
| 6-8:coNr 2 | -11·00 (2·85) | <·001** |  | -16·33 - -5·64 |
| 9-11:coNr 2 | -9·27 (2·84) | <·001** |  | -14·58 - -3·91 |
| 12-14:coNr 2 | -13·46 (2·98) | <·001** |  | -19·04 - -7·83 |
| 15-17:coNr 2 | -11·38 (3·07) | <·001** |  | -17·10 - -5·55 |
| 18-24:coNr 2 | 12·04 (2·86) | <·001** |  | -17·38 - -6·66 |
|  |  |  |  |  |
| 0-2:coNr 3 | -4·84 (2·52) | ·056 |  | -9·54 - -0·12 |
| 3-5:coNr 3 | -4·08 (3·05) | ·181 |  | -9·76 – 1·60 |
| 6-8:coNr 3 | -3·30 (2·62) | ·209 |  | -8·19 – 1·60 |
| 9-11:coNr 3 | 0·44 (2·80) | ·873 |  | -4·77 – 5·68 |
| 12-14:coNr 3 | 3·15 (3·12) | ·313 |  | -2·66 – 9·00 |
| 15-17:coNr 3 | 3·76 (3·35) | ·262 |  | -2·47 – 10·01 |
| *Model Information* |  |  |  |  |
| AIC | 1268·82 | .. | .. | .. |
| BIC | 1346·77 | .. | .. | .. |
| Number of observations | 219 | .. | .. | .. |
| Number of Groups | 127 | .. | .. | .. |

SE= Standard Error, AIC= Akaike Information Criterion, BIC= Bayesian Information Criterion, 95%CI= 95% Confidence Interval. Note: the fixed-effect model matrix was rank deficient, leading to the removal of the last column ‘18-24:coNr 3’ because it did not provide any additional information. The likelihood for the null model was -695·56, and -611·41 for the full model (M3). The likelihood ratio test indicated a significant improvement in fit (χ²(20) = 168·29, p < ·001).

1. Rosenbek, J.C., et al., *A penetration-aspiration scale.* Dysphagia, 1996. **11**(2): p. 93-8.

2. Zang, J., et al., *Flexible endoscopic evaluation of swallowing in children with type 1 spinal muscular atrophy.* Eur Arch Otorhinolaryngol, 2023. **280**(3): p. 1329-1338.

3. Schober, P., C. Boer, and L.A. Schwarte, *Correlation coefficients: appropriate use and interpretation.* Anesthesia & analgesia, 2018. **126**(5): p. 1763-1768.
